# ADEPT: a domain independent sequence alignment strategy for gpu architectures

**DOI:** 10.1186/s12859-020-03720-1

**Published:** 2020-09-15

**Authors:** Muaaz G. Awan, Jack Deslippe, Aydin Buluc, Oguz Selvitopi, Steven Hofmeyr, Leonid Oliker, Katherine Yelick

**Affiliations:** Lawrence Berkeley National Laboratory, 1 Cyclotron Road, Berkeley, USA

**Keywords:** Bioinformatics, GPU, Alignment, Protein, DNA

## Abstract

**Background:**

Bioinformatic workflows frequently make use of automated genome assembly and protein clustering tools. At the core of most of these tools, a significant portion of execution time is spent in determining optimal local alignment between two sequences. This task is performed with the Smith-Waterman algorithm, which is a dynamic programming based method. With the advent of modern sequencing technologies and increasing size of both genome and protein databases, a need for faster Smith-Waterman implementations has emerged. Multiple SIMD strategies for the Smith-Waterman algorithm are available for CPUs. However, with the move of HPC facilities towards accelerator based architectures, a need for an efficient GPU accelerated strategy has emerged. Existing GPU based strategies have either been optimized for a specific type of characters (Nucleotides or Amino Acids) or for only a handful of application use-cases.

**Results:**

In this paper, we present ADEPT, a new sequence alignment strategy for GPU architectures that is domain independent, supporting alignment of sequences from both genomes and proteins. Our proposed strategy uses GPU specific optimizations that do not rely on the nature of sequence. We demonstrate the feasibility of this strategy by implementing the Smith-Waterman algorithm and comparing it to similar CPU strategies as well as the fastest known GPU methods for each domain. ADEPT’s driver enables it to scale across multiple GPUs and allows easy integration into software pipelines which utilize large scale computational systems. We have shown that the ADEPT based Smith-Waterman algorithm demonstrates a peak performance of 360 GCUPS and 497 GCUPs for protein based and DNA based datasets respectively on a single GPU node (8 GPUs) of the Cori Supercomputer. Overall ADEPT shows 10x faster performance in a node-to-node comparison against a corresponding SIMD CPU implementation.

**Conclusions:**

ADEPT demonstrates a performance that is either comparable or better than existing GPU strategies. We demonstrated the efficacy of ADEPT in supporting existing bionformatics software pipelines by integrating ADEPT in MetaHipMer a high-performance denovo metagenome assembler and PASTIS a high-performance protein similarity graph construction pipeline. Our results show 10% and 30% boost of performance in MetaHipMer and PASTIS respectively.

## Background

Sequence alignment lies at the core of most bioinformatics applications. Aligning two sequences determines a degree of similarity which may yield homology of the proteins or genes and their functional information. Local sequence alignment has been used in de novo sequence assembly to determine how different regions of a genome are connected [1, 2] and for determining overlapping regions of long reads [3]. It has been used to determine conserved regions in proteins and genes, which has applications in evolutionary biology and functional genomics [4].

Smith-Waterman is a sequence alignment algorithm that scores all possible local alignments between two sequences using a dynamic programming method and outputs the optimal alignment [5]. Gotoh modifications enable the algorithm to account for gap openings and extensions [6]. Because of its exhaustive nature, the Smith-Waterman algorithm has a worst-case time and space complexity of *O*(*nm*) where *n* and *m* represent the lengths of two sequences to be aligned. Its quadratic time complexity makes performing large number of alignments or aligning long sequences time consuming. As a solution, heuristic based strategies were presented in the form of BLAST and Gapped BLAST which speed up the process considerably with the trade-off being an approximate solution [7, 8].

In this paper we present ADEPT, a novel domain independent sequence alignment strategy for GPU architectures and demonstrate it by implementing a GPU-accelerated complete Smith-Waterman algorithm for the use case of pairwise sequence alignments. ADEPT derives its performance from architecture specific optimizations and is performant regardless of the type of sequence. Our analysis shows that ADEPT out-performs similar CPU approaches and either closely matches or out-performs domain specific existing GPU approaches. ADEPT provides an added advantage of built in capability of scaling across multiple GPUs with minimal effort from the developer. It can effectively be used as a drop-in replacement for CPU libraries. For the rest of this paper, the acronym ADEPT will be used interchangeably for the ADEPT-based implementation of the Smith-Waterman algorithm and the proposed strategy in general.

### Prior work

**Fig. 1:**
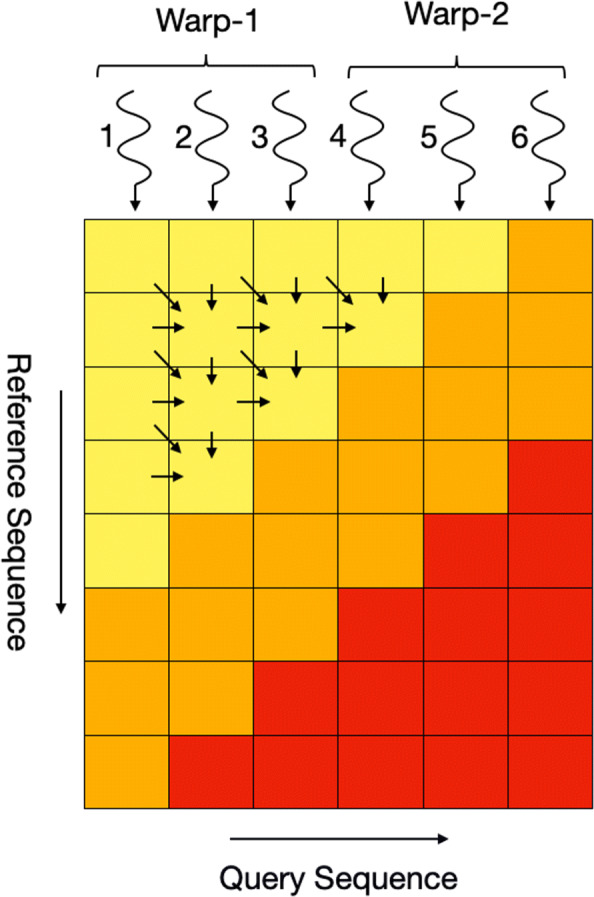
Arrow heads point towards the cell being computed while arrow tails lie in the cells that computation depends upon. At a given time, only the cells along the anti-diagonal can be calculated by the algorithm in parallel. Three shaded region show different parts of the algorithm: in the yellow region the parallelism increases with each iteration, in the orange region it remains constant, while in the red region it starts to decrease

**Fig. 2:**
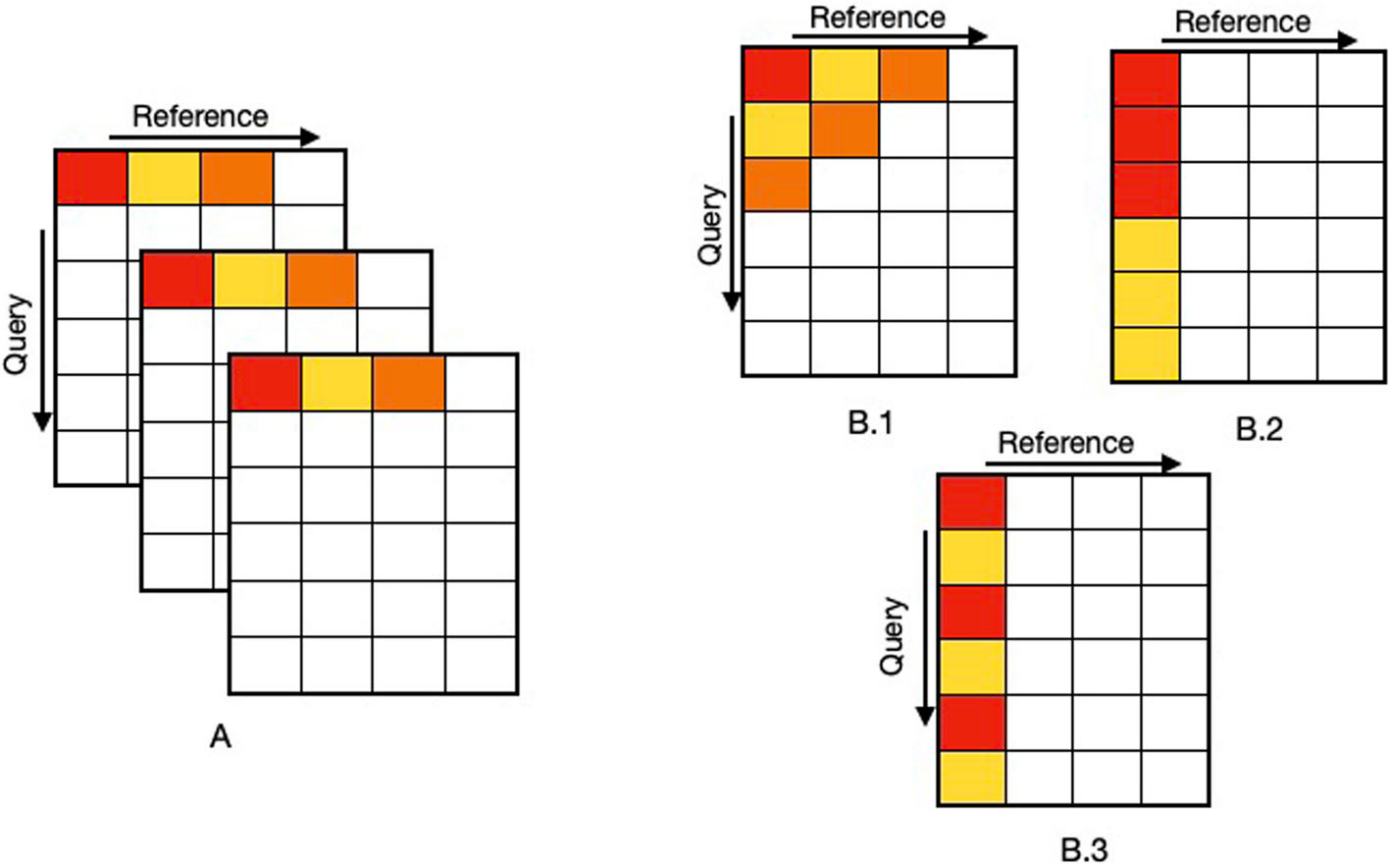
In this figure, similarly colored boxes are computed in parallel. **A)** Shows the typical inter task parallelism strategy where multiple DP tables are constructed in parallel. **B**.1) Shows the wavefront parallelism strategy. **B**.2) Shows Rognes’ Intra task approach. **B**.3) Shows Farrar’s striped approach

With the introduction of multi-core and GPU devices, multiple parallel strategies for exploiting modern architectures were introduced. Parallelizing the Smith-Waterman algorithm is particularly challenging because of the inter-cell dependencies in the dynamic programming matrix [9]. Computation of each cell depends on the cell above, diagonally above and on the left, as shown in Fig. 1. These strategies can be classified into two major categories 1) Intra-Task Parallelism, where fine-grained parallelism is introduced for aligning two sequences and 2) Inter-Task Parallelism, where each sequence alignment is considered as an independent task and performed in parallel. The first category includes wavefront parallelism, where the cells along the anti-diagonals can be computed in parallel as shown in Fig. 2 (B.1). This strategy has been implemented for CPU SIMD units by Wozniak and a GPU version was implemented in CUDAlign, which targets the use case of Megabase (DNA) alignment where the sequences to be aligned are very long [10, 11]. Another intra-task approach of computing the cells along the query sequence (as shown in Fig. 2(B.2)) was introduced and implemented by Rognes et. al [12] for CPU SIMD units, the same colored boxes show how the regions of table are mapped to SIMD units. Currently, there is no known GPU implementation for this method. In 2006 Michael Farrar introduced another intra-task approach in the form of the Striped Smith-Waterman algorithm for CPU SIMD units, as shown in Fig. 2 (B.3) [13]. This strategy proposed computing the cells in a striped manner parallel to the query sequence, while ignoring certain dependencies and making up for that by including an error correction loop for ensuring correctness. There is also no known GPU implementation for the Striped strategy.

Inter-task parallelism strategies translate to embarrassingly parallel approaches that compute multiple alignments in parallel. One such implementation, which has been discussed by Rognes [9], involves mapping a sequence alignment per processing unit such that multiple DP tables are constructed in parallel (for different alignments) and cells of each are computed concurrently (see Fig. 2 (A)). The basic idea of this strategy, accompanied by some device and application specific optimizations, has yielded good results for GPU developers. For instance, CUDASW [14] utilizes a hybrid method consisting of wavefront parallelism and an embarrassingly parallel method to perform protein sequence alignments. Depending upon the sequence lengths CUDASW switches algorithms between wavefront and inter-task approaches. It also utilizes a query profile, a common optimization strategy for protein database alignments to minimize memory accesses [13]. Another protein specific GPU implementation has been discussed in [15], which also implements the embarrassingly parallel approach where each CUDA thread aligns a protein sequence from a database, with the query protein. The authors used optimizations to better exploit GPU architectures, such as ensuring that consecutive threads perform almost same amount of work and clustering together data accesses to minimize cache misses. A more recent approach in the same category is GASAL2 [16], which implements an inter-task parallelism approach to perform pairwise DNA alignments. GASAL2 targets the use-case of short read alignments, and has been optimized for DNA sequences only. It uses domain specific optimizations such as encoding DNA bases using only four bits to maximize memory bandwidth utilization. GASAL2 is the fastest GPU implementation for aligning DNA short reads [16]. However, GASAL2 does not support protein alignment.

### Problem statement

A very common scenario in bioinformatics applications requires pairwise sequence alignments where one-to-one alignments are performed between two given sets of sequences [1, 16]. This problem is different than all-to-all approaches presented in [14, 15] and requires a different approach. A typical all-to-all approach is that of a protein database where each query sequence is compared against all the possible targets in the reference set [13], which would lead to *NM* total alignments, where *N* and *M* are sizes of query and reference sets respectively. One-to-one alignment deals with aligning only those sequences which are present at same indices in two sets of sequences, i.e. given that total sequences in query and reference sets are *N*, then total alignments would also be *N*.

Use cases of one-to-one pairwise alignments are quite common in short-read DNA mapping [17, 18] and in DNA assemblers [19, 20]. In these cases all-to-all alignments are not required; in fact selected sets of reads are aligned with selected sets of target candidates, which can be achieved using one-to-one pairwise alignment.

Similarly, one-to-one pairwise alignments play an important role in inferring homologous proteins. The detection of homologous proteins is fundamental to several applications such as functional annotation (assigning functions to unknown proteins), gene localization (identifying genes that are of a particular functionality of interest), or identifying protein families (the proteins that descend from a common ancestor). For example, a common method for the identification of protein families is (i) to first perform a similarity search [21, 22] in a filtered set of amino acid sequence pairs by running a batch of pairwise local or global alignments, (ii) then use this alignment information to form a protein similarity network, and (iii) finally cluster [23–25] this network to discover the protein families. Here, the information obtained from pairwise alignments include metrics such as identity, score, coverage, etc. and they are used in determining the structure of the protein similarity network. The batch pairwise alignment usually constitutes the most time-consuming step and it is important for this step to benefit from accelerators to enable identification of families in large protein datasets.

However, the available GPU implementations employ methods which are either domain specific, such as query profile construction for protein database search [14] and use of bit-encoding for DNA sequences [16], or use-case specific, such as the Megabase use-case [11]. These methods do not allow for performance portability across all domains and applications of bioinformatics and are highly specialized. By contrast, CPU SIMD libraries like Seqan [26] and SSW-Library [27] are domain-independent and derive their performance by better exploiting the hardware architecture rather than relying on domain specific optimizations.

This leaves a gap for a parallel strategy that leverages a GPU’s hardware to derive performance rather than relying on application specific approaches. Such a method could enable offloading of sequence alignments to the GPU regardless of the type of sequence (Protein or DNA). Such libraries are widely available for CPUs and have enabled the development of numerous tools due to their generic nature. In an attempt to mitigate this gap, in this paper we introduce ADEPT, a parallelization strategy that can exploit a GPU’s architecture for performance, to provide a consolidated GPU-accelerated sequence-alignment library.

### Smith-waterman algorithm

Consider two sequences *Q* and *R* to be aligned; *Q* is a query sequence represented as *Q*={*q*_1_,*q*_2_,*q*_3_,,,*q*_*n*_} and *R* is a reference sequence represented as *R*={*r*_1_,*r*_2_,*r*_3_,,,*r*_*m*_}, where *n*=|*Q*| and *m*=|*R*|. In this paper we consider the case of one-to-one alignments where, given two sets of sequences, *A* and *B*, each sequence in set *A* will be aligned to one sequence in set *B* located at the same index. The total number of sequences in set *A* is equal to the number of sequences in set *B*.

Given the sequences *Q* and *R*, Smith-Waterman with Gotoh scoring computes three scoring tables *E*, *F* and *H*, following the equations below:

1 $$ E_{ij}=Max (E_{i,j-1}+G_{ext}, H_{i,j-1}+G_{init})  $$

2 $$ F_{ij}=Max (F_{i-1,j}+G_{ext}, H_{i-1,j}+G_{init})  $$

3 $$ H_{ij}=Max (E_{i,j},F_{i,j},H_{i-1,j-1}+S(q_{i},r_{i}),0)  $$

In the above equations, matrix *E* and *F* are used for keeping track of gap insertions in reference and query sequences. A gap insertion in query sequence can be seen as a deletion in reference sequence or vice versa. Gap insertion/deletion or collectively known as *indels* enable accurate alignment of two sequences even if they are not of equal lengths. Without matrices *E* and *F* it is not possible to penalize gap insertion separately. Matrix *H* keeps track of alignment extensions. *G*_*init*_ is the gap initialize penalty, *G*_*ext*_ is the gap extend penalty and *S*(*a*,*b*) is the match or mismatch score based on how closely characters *a* and *b* match. The algorithm starts by initializing the first row and column of tables *E*, *F* and *H* with zeroes. The first phase involves computing each cell of table *H* with the help of tables *E* and *F*, using the above equations. While scoring the table *H*, information is maintained to keep track of the highest scoring cell (from Eq. 3) in each iteration. After populating the scoring matrix *H*, the second phase involves obtaining the highest scoring cell *h*_*i*,*j*_ from table *H*. Indices *i* and *j* of this cell indicate the ending location of the optimal alignment. The third phase involves performing a traceback step starting from the highest scoring cell and following the optimal path until a cell with a score of zero is reached; this gives the starting location of the optimal alignment.

### Graphics processing units

Graphics Processing Units (GPUs) were introduced as dedicated graphics processing devices, but with the development of advanced programming tools and improvement in GPU hardware, they have rapidly emerged as accelerators of choice across the High-Performance Computing community. A typical CPU-GPU computing setup involves selecting a computationally intensive portion of an application and offloading it to GPU. This involves offloading the data to the GPU’s Global Memory, launching a kernel to run on the GPU, and then moving the results back from the GPU to the CPU. CPU and GPU communication happens via a PCI express connection.

GPU hardware consists of multiple Streaming Multiprocessors (SM), where each SM contains multiple Floating-Point units, Integer operation units and in more recent devices Tensor operation units. All the cores collectively provide GPUs with their massively parallel nature. For instance, NVIDIA’s V100 GPU contains a total of 80 SMs and 64 FP32 units giving a total of 5,376 computing cores. GPUs traditionally have an on-chip memory resource, termed Shared Memory, and off-chip Memory or Global Memory [28]. Typically, Global Memory is of the order of gigabytes and is the primary location where data is offloaded from the CPU for processing. In comparison, Shared Memory is quite scarce and is usually on the order of kilobytes. A portion of the shared memory can be configured to be used as an L1 cache to improve compiler aided optimizations or be used as shared memory or a programmer controlled cache. An L2 cache is also present to improve memory re-use but is not controlled by the programmer. To understand typical sizes of these memories, consider the NVIDIA V100 GPU, which has 16GB of Global Memory, 96KB of Shared Memory/L1 cache per SM and 6144KB of L2 cache in total. The register file size for each SM is 256 KB. A better use of a GPU’s memory hierarchy can yield considerably better application performance [29].

### CUDA platform overview

CUDA is a parallel programming platform which enables the use of CUDA-enabled GPUs for general purpose computing. CUDA provides lower level software access to the computational elements of GPUs and enables a programmer to write kernels for offloading computational load to GPUs. The CUDA programming model provides two levels of parallelism in the form of a grid of CUDA blocks, where each CUDA block consists of multiple threads. CUDA Threads are the basic computational unit; each thread can be identified by a unique thread id and a block id representing its parent block. Inter-thread communication can take place either via the Shared Memory or using register-to-register data transfers. Inter-block communication can happen only via the Global Memory. In NVIDIA hardware, the threads of a block are scheduled on to the SMs in groups of 32 known as warps. Depending upon the resource availability, multiple warps may be scheduled on the same SM.

## Implementation

### Initialization

**Fig. 3:**
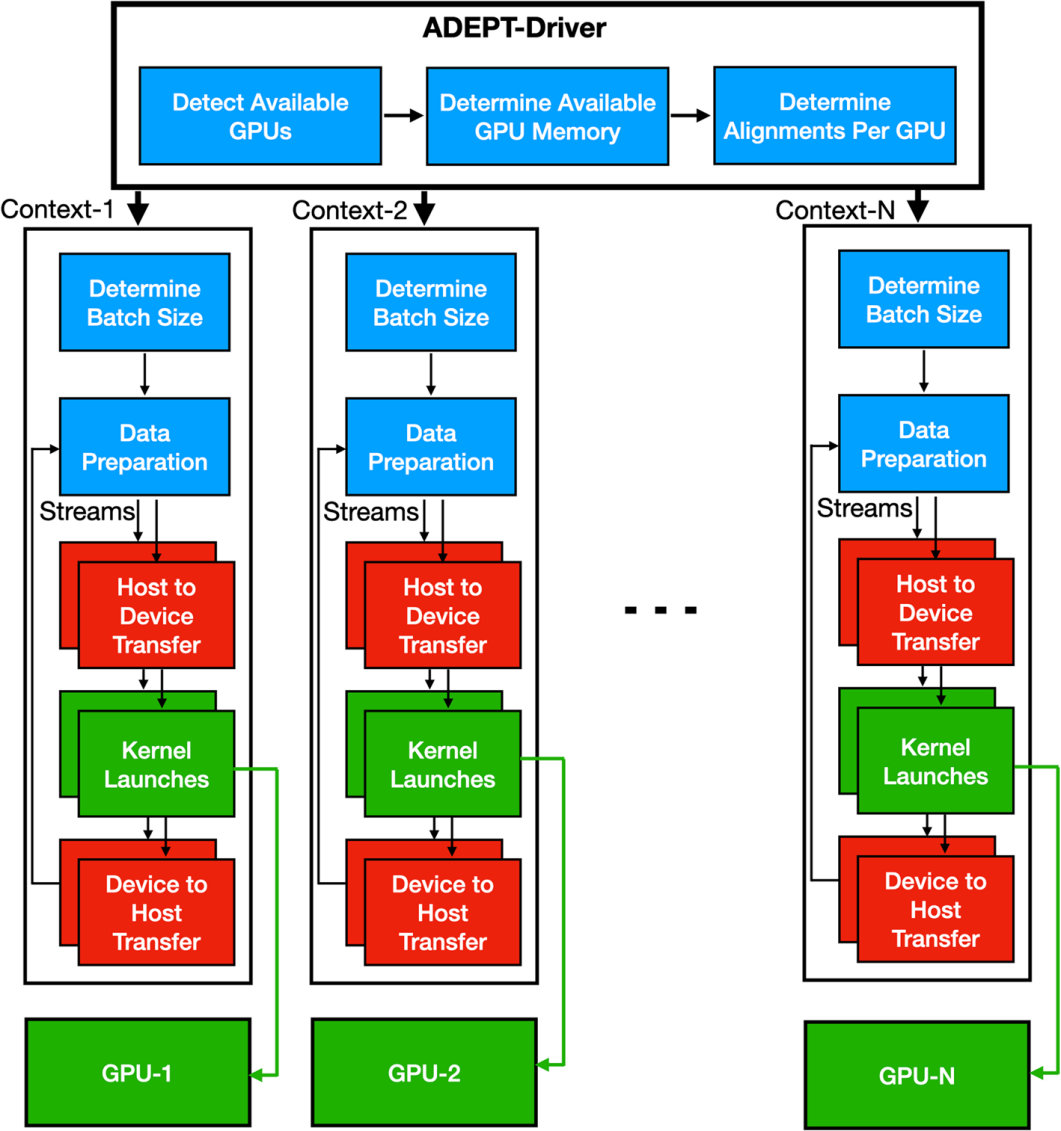
The overall pipeline of the ADEPT strategy. ADEPT’s driver detects all the available GPUs and their available memory. Based on this, it determines the amount of work that can be dispatched to each GPU. A separate CPU context is created for each GPU. On the CPU side, the batch size is determined based on the GPU’s available memory or the batch size can also be fixed by the user. To overlap the data preparation step, data transfers and the GPU computation, multiple CUDA streams are used. Immediately after making the GPU to CPU transfer call, the CPU returns to the data preparation step while kernel execution is still going on at the GPU side. This asynchronous behavior overlaps the CPU and GPU parts of the computation

Our ADEPT implementation has two parts: a driver and a kernel. The driver initializes the GPU memory, packs the sequences into batches, and once enough sequences are available to saturate the GPU global memory, transfers all this data to the GPU. Additionally, the driver also detects different GPUs available on the node and balances the amount of work across all the available GPUs as shown in Fig. 3.

Batched sequences are stored in two arrays, one for query sequences and the other for reference sequences. The number of sequences in the query and reference arrays are the same, and sequences located at the same indices are aligned with each other. For instance, if there are N query sequences and N reference sequences, a total of N alignments will be performed. Each alignment is mapped to a unique CUDA block. Then, inside each CUDA block a more fine-grained approach is implemented. From here on, all the implementation details are per-block and the same algorithm is replicated across each CUDA block.

### Tracking inter-thread dependencies

As discussed in the Background section, to compute each cell *H*_*ij*_ of the dynamic programming table *H*, cells *H*_*i*−1,*j*_, *H*_*i*,*j*−1_, and *H*_*i*−1,*j*−1_ need to have been computed. Because of this aspect of the algorithm, parallelism is restricted only along the ant-diagonal of the matrix as shown in Fig. 1. It can be further observed in the figure that first the amount of parallel work increases as the algorithm progresses, then remains constant for some iterations, and finally starts to decrease near the bottom right triangle of the matrix (shown in red). The maximum number of cells that can be calculated in parallel at any given time is equal to the length of the shorter of the two sequences. This poses the unique challenge of keeping track of dependencies for different cells and masking out the threads for which dependencies are not ready.

To calculate the scoring table *H*, we start by assigning one CUDA thread per column (as in Figure 1) such that it computes all the scoring cells within that column. Here we assume that of the two sequences being aligned, the longer sequence is mapped along the column and is referred to as *R*. As discussed before, because of dependencies, not all the threads can progress together. To tackle this problem, we introduce a *Binary Masking Array* (BMA) for masking out threads in each iteration for which dependencies are not ready. The *BMA* has a length *b*, where *b* is equal to 3∗|*Q*| and *BMA* is initialized as:

4 $$ x_{i}=\left\{ \begin{array}{cc} 0,& \text{if} (i < |Q|) or (i > 2*|Q|)\\ 1, & \text{otherwise} \end{array}\right.  $$

In the above equation, *x*_*i*_ is the *i*^*th*^ element of BMA. The number of threads that need to track their state is equal to the size of query sequence. Since each thread needs to track its state in three different phases of algorithm, the length of *BMA* is fixed to three times the size of query sequence.

**Fig. 4:**
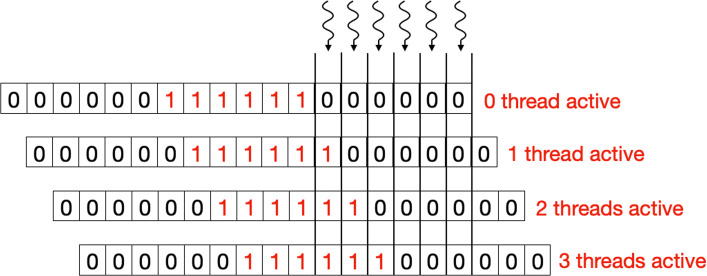
The zero/one array in this figure represents the Binary Masking Array (BMA) in the yellow region of the algorithm for the DP table in Fig. 1. With each iteration the array shifts to the right, activating one additional thread per iteration (given that the condition *C* is valid)

Figure 4 shows the BMA array for a query of length 6. Here, each thread keeps track of an element in BMA. After each iteration, if the algorithm is in the yellow region (see Figure 1), the array shifts to right, activating one more thread. Condition *C* is used to keep track of the region which the algorithm is in.

5 $$ C = I < |Q| or I >= |R|  $$

**Fig. 5:**
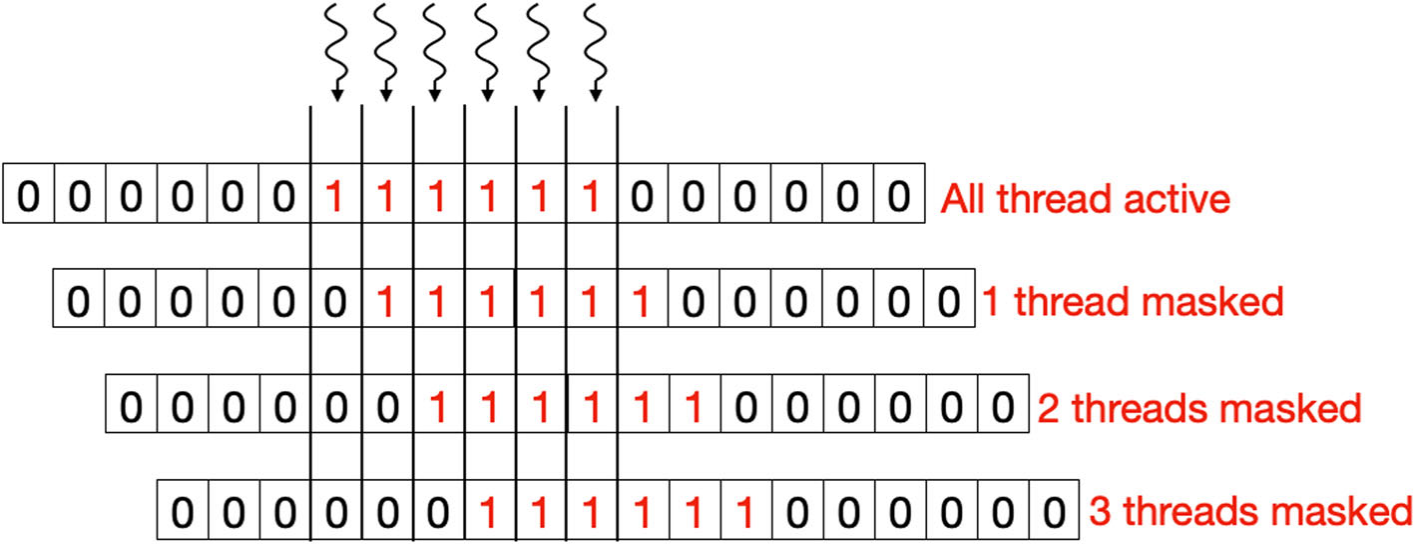
The zero/one array in this figure represents the Binary Masking Array (BMA) while the algorithm is in the red region for the DP table in Fig. 1. With each iteration the array shifts to the right, deactivating one additional thread per iteration (given that the condition *C* is valid)

In the above equation, *I* is the iteration number, which also corresponds to the diagonal being computed. It can be observed in Fig. 4 that initially no CUDA thread was active and as the algorithm progresses more and more threads are activated to perform the work. Once the algorithm reaches the orange region, the condition *C* becomes false and the array stops shifting until the algorithm enters the red region. Here again the array starts shifting to the right (as shown in Fig. 5) with each iteration, but this time threads are getting masked out with each iteration because of the decreasing diagonal size, this can be observed in Fig. 1. Pseudo code in Algorithm 1 shows the usage of BMA in keeping tracking of algorithm’s state.

### Dynamic programming table storage and memory access issues

To compute the highest scoring cell a pass over the complete table H is required. Since the total number of cells that are computed in the table H are *n*∗*m*, if we use 2 bytes to store each cell, storing the complete dynamic programming table in memory requires *N*∗(2∗*mn*) bytes. Where *N* is the size of a batch. This yields a total global memory requirement of several hundred GBs for a million alignments, and even top of the line GPUs have global memory of only a few GBs.

**Fig. 6:**
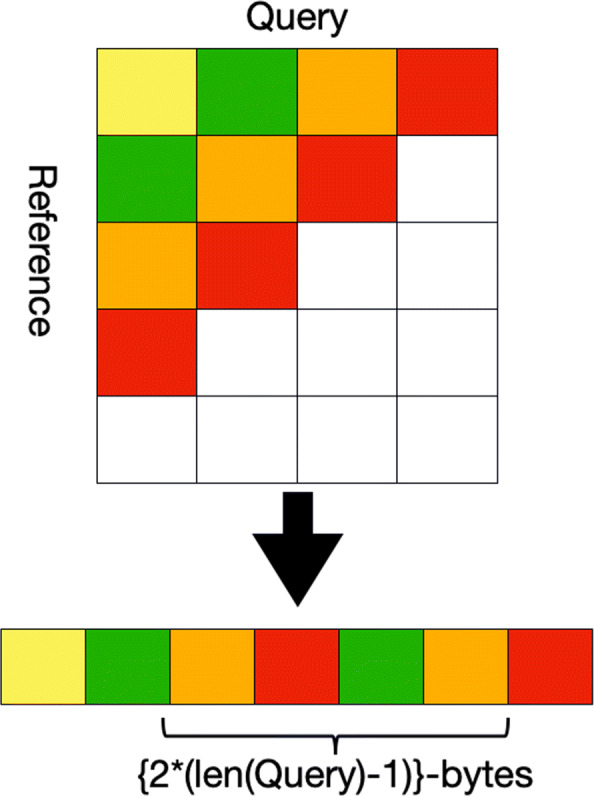
When reading or writing along the anti-diagonals of a matrix that has been stored in a column major way, consecutive elements of an anti-diagonal are placed 2∗(|*Q*|−1) bytes apart. This leads to un-coalesced global memory accesses in GPU

Apart from the storage size of the dynamic programming table, another challenge that occurs often on GPUs is that of non-coalesced global memory accesses. The Global memory accessed by threads of a CUDA warp is bundled into the minimum number of cache loads, where L1 cache line size is 128 bytes. Thus if the two threads are accessing a memory location that is more than 128 bytes apart, the memory accesses will be un-coalesced. It can be observed in Fig. 6 that while performing a write back to global memory to store the table H, memory accesses are about 2∗(*n*−1) bytes apart, which can be more than 200 bytes apart if *n* is larger than 100.

It can be seen in Fig. 1 that to compute a given anti-diagonal using the proposed parallel approach only the two recent most anti-diagonals are required. Apart from computing the maximum scoring cell at the end of scoring phase, there is no reason for storing the complete scoring matrix, except for the two most recent diagonals. As a solution to the problem of computing the maximum scoring cell, we modified our implementation so that each thread can maintain a running maximum score for the column it has been assigned; this can be kept in the thread’s register. Thus, we can effectively discard the scoring matrix beyond the two most recent diagonals. Since this requires storing only a portion of the matrix, this can be done inside thread registers thus avoiding the problem of non-coalesced memory accesses.

Once all the cells have been computed we use CUDA’s warp shuffle intrinsics to implement a block-wide reduction for obtaining the highest scoring cell as shown in Algorithm 1. Our implementation of block-wide reduction has been adopted from NVIDIA’s own reduction method [30] with modifications introduced to obtain the indices of the highest scoring cell along with the score.


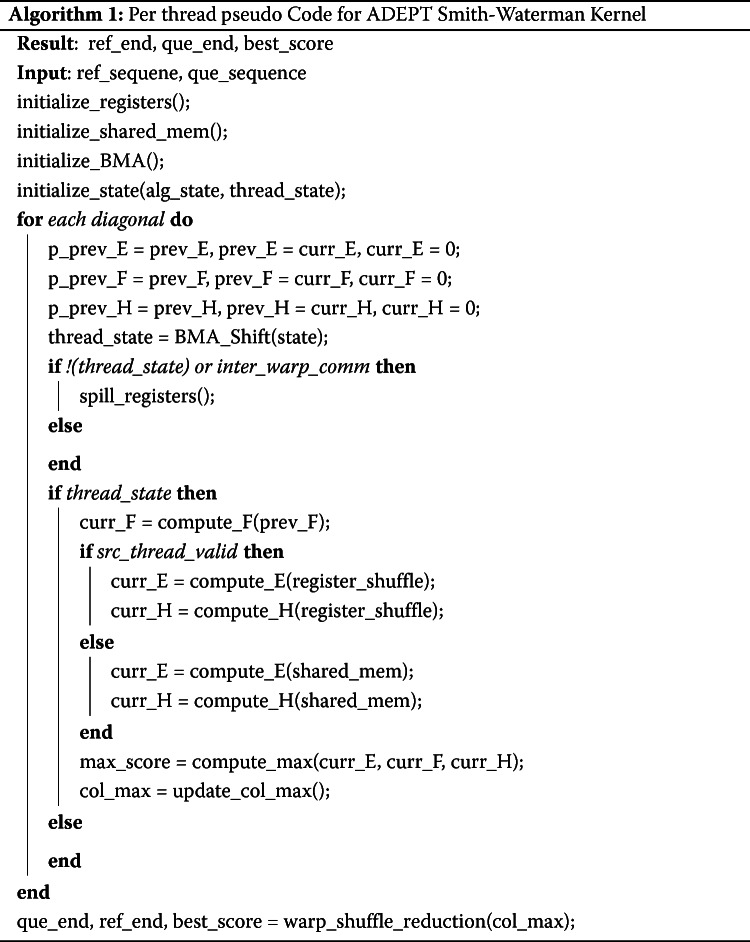


### Efficient inter thread communications

Figure 1 shows the mapping of CUDA threads to columns of the scoring matrix. It can be observed in the figure that because of the cell-dependencies there is inter-thread communication required between the two consecutive threads. For a thread j to compute the cell *H*_*i*,*j*_ it requires values from cells *H*_*i*−1,*j*_, *H*_*i*,*j*−1_ and *H*_*i*−1,*j*−1_. In the figure it can be observed that the cell *H*_*i*−1,*j*_ is computed by thread *j* while the other two cells are computed by thread *j*−1. For this inter-thread transfer we explored two methods of data sharing between threads i.e. communication using shared memory and register-to-register memory transfer.

CUDA’s warp shuffle intrinsics allow threads to perform direct register-to-register data exchange without performing any memory loads and stores, while use of shared memory involves going through the on-chip shared memory. Due to much faster performance we opted for the register-to-register data exchange method.

However, register-to-register transfers are only allowed among the non-predicated threads of the same warp. This introduces several edge cases, for example in the CUDA platform where a warp is 32 threads wide, a communication between thread (32∗*q*)−1 and 32∗*q* (where *q*>0) would not be possible through register-to-register transfers because these do not belong to same warp. For instance, in Figure 1, threads 3 and 4 belong to different warps (assuming that a warp is three threads wide), so they cannot communicate via the register exchange method. For such cases, the last thread of each warp spills its registers to the shared memory every iteration so that first thread of the next warp can retrieve that data.

Similarly, while computing the scores for cells *H*_*m*,*j*_, the threads j-1 would have been predicated (in Fig. 1, each thread is masked after it has computed the last cell of the column it is assigned to) and a register-to-register transfer would not be possible. To cater for these edge cases, we use shared memory arrays to spill the values of thread registers whenever such edge cases occur. Using the BMA method discussed in previous section, it becomes quite straight forward to determine if a certain thread will be predicated in the next iteration so that its registers are timely spilt to shared memory and then any dependent threads can access the required values from shared memory.

Using the above method provides fast inter-thread communication along with freeing up significant amount of shared memory, which helps improve GPU utilization and also helps avoid shared memory bank conflicts. A bank conflict occurs when multiple threads access same bank of shared memory, this enforces sequential access to that portion of memory and results in performance degradation.

An overall step by step kernel pseudo code for forward phase has been provided in Algorithm 1.

### Reverse scoring

The third phase of the Smith-Waterman algorithm involves performing a traceback starting from the highest scoring cell and ending when the score drops to zero or the top left end of the matrix is reached. This requires maintaining the traceback pointers, which can be stored in the form of two matrices, one for storing the indices of the query sequences and the other for storing the indices of the reference sequences. However, storing these matrices yields two sets of challenges. First, the amount of memory required to store traceback matrices equals 2∗*N*∗(*n*∗*m*), which can be several hundred GBs when *N* is close to a million alignments and unlike the scoring phase we cannot discard parts of the traceback matrices because that may lead to missing optimal alignments. The second challenge is that of un-coalesced memory accesses, as mentioned before. The write-back to the traceback matrices occurs along the anti-diagonals and since the matrices are laid down in the global memory in row-major indexing, this leads to un-coalesced memory accesses as shown in Fig. 6.

However, in most of the practical Smith-Waterman applications, complete alignment details are rarely required. The majority of the applications only require the optimal alignment score and the optimal alignment start and end indices [1, 13, 27]. Details of insertions and deletions are typically not required when the Smith-Waterman algorithm is being used as a part of a computational pipeline, in particular for the case of pairwise alignments. Considering this practical reason, rather than performing a detailed traceback, we use a reverse scoring phase.

#### Reverse scoring phase

To obtain the start positions of the alignment we make use of the symmetric nature of the optimal alignment. An optimal alignment is symmetrical i.e. scoring two sequences forward or with their directions reversed yields the same optimal alignment.

**Fig. 7:**
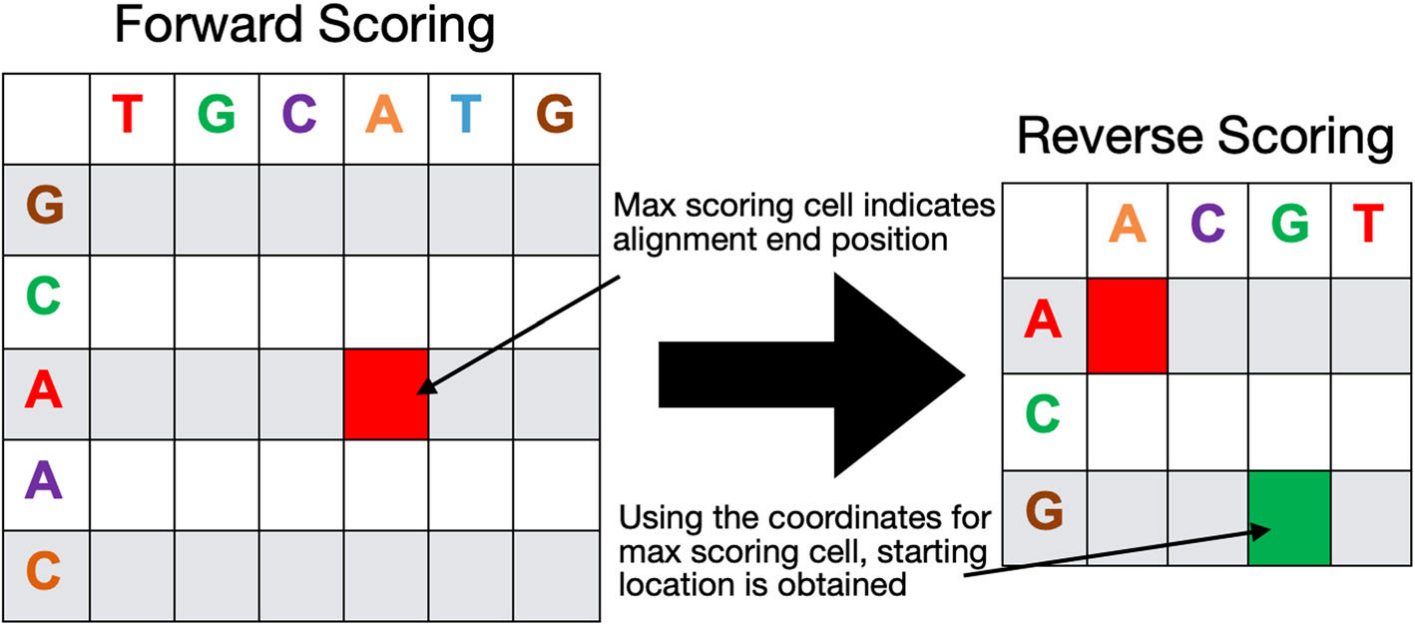
Sequences are flipped in the reverse scoring phase and the same kernel is used as the forward phase. The highest scoring cell in the reverse phase then provides the start location of alignment

For reverse scoring, we make use of this property as previously done in [27] and compute a reverse scoring matrix with both the sequences flipped from the indices of the highest scoring cell in the forward scoring matrix. When scoring in reverse, the highest score will correspond to the same alignment as the one in the forward scoring phase as shown in Fig. 7.

Using the indices of the highest scoring cell in the reverse scoring phase, we can compute the start index of alignment. Using the reverse scoring phase enables us to avoid storing traceback matrices and helps free up GBs of space. The reverse scoring kernel follows the same implementation as the forward scoring kernel that has been shown in Algorithm 1, hence we re-use that implementation by providing flipped sequences at the input. It must be noted that the reverse scoring matrix in most of the cases ends up having less total work because of known end positions.

### Support for protein alignment

Thus far, ADEPT has not required any domain specific optimizations, and the Smith-Waterman implementation discussed above has been oblivious of the types of sequence.

The difference between aligning protein sequences and DNA sequences is between the scoring methods. When aligning DNA sequences, if two of the same nucleotide bases align, that is considered a match and a fixed match score is used for computing the total score; similarly if the bases do not match, a mismatch score is used instead. When aligning protein sequences, two aligning amino acids need to be scored based on their chemical similarity. Similarity scores for all possible comparison of amino acids are characterized and available in the form of a scoring matrix [31]. Instead of a match/mismatch score for protein sequencing a user needs to provide a scoring matrix.

Since a scoring matrix needs to be accessed very frequently, in our implementation we move the static scoring matrix to the GPU’s shared memory to reduce the overhead associated with multiple accesses. For simplifying the scoring matrix lookups and minimizing shared memory usage, we use a decoding matrix to index into the scoring matrix. Typically, the scoring matrix is indexed by the amino acid characters, which leads to large amount of memory being reserved for the matrix. In this implementation, we first index a with the ASCII code associated with the amino acid character to retrieve an encoded index, which is then used to access the scoring matrix.

Underlying kernel for protein and DNA alignment still remains the same, for protein alignment the only difference is that instead of a match/mis-match score and similarity score is obtained from the scoring matrix, everything else remains the same as in Algorithm 1.

In order to make the switch between protein kernel and DNA kernel easy for the user, we provide two different kernels for protein alignment and DNA alignment. The DNA kernel accepts match, mismatch, gap open and gap extend scores at input while the protein kernel accepts a scoring matrix along with gap open and gap extend scores at compile time.

### Multi-GPU asynchronous pipeline

In a typical CPU-GPU setup, the CPU prepares a batch of data that is offloaded to a GPU and launches a GPU kernel to process that data; once the data is processed, the results are moved back to CPU. However, with the evolution of GPU technology, a widespread adoption of GPUs has taken place, and instead of having one GPU per node, a typical GPU system has several GPUs on each node. For instance, the Summit supercomputer [32] has six GPUs per node and the upcoming Perlmutter supercomputer is planned to have four GPUs per node [33]. This calls for a software setup which would determine the type and memory capacity of each GPU on a node dynamically and divide the work among all GPUs accordingly. As a solution, ADEPT contains a *driver* component which manages all the communication, load balancing and batch size determination for the GPU kernels while keeping the developer oblivious of these intricacies.

ADEPT’s driver gathers hardware information about all the GPUs installed on a node and then divides the work equally among them. A separate context is created for each GPU where a unique CPU thread is assigned to a particular GPU. This CPU thread divides the total computational load into smaller batch sizes depending on the memory capacity of the GPU assigned to it. Each batch is then prepared and packed into a data structure which is then passed to a GPU kernel call. Using CUDA streams, the GPU kernel call and the data packing stage are overlapped so that CPU and GPU work can be carried out in parallel. An overview of ADEPT’s design can be seen in Fig. 3.

ADEPT’s driver makes it easier for the developers to integrate ADEPT in high performance bioinformatics software pipelines by reducing the complexities of dealing with multiple GPUs, and requiring just one call to the driver function. Effectively, making ADEPT a drop-in replacement for existing CPU libraries, whereas existing GPU libraries require significant amount of work in order for them to be included in an existing software pipeline.

## Results

We evaluate the performance of ADEPT against two of the popular CPU libraries which can perform both Protein and DNA alignments. Among existing GPU implementations we chose GASAL2 for comparison because it is the fastest known GPU library for performing DNA pairwise alignments [16]. The only known GPU alignment library that can perform pairwise protein alignments is NVBIO [34], so we compared ADEPT against the protein alignment tool of NVBIO. The libraries we are evaluating ADEPT against include:

***SSW-library*** Striped Smith Waterman or SSW-Library [27] is an implementation of Farrar’s algorithm [13] and is one of the fastest known CPU implementations of the Smith-Waterman algorithm. SSW-Library leverages the CPU’s vector instruction set.

***Seqan library*** Seqan Library is a widely used CPU sequence alignment library [26]. For this paper we use the Seqan test suit developed by the authors of the Seqan library [35]. For these experiments we made use of *align_bench_par* program as it performs pairwise alignments. We used the build option for AVX2 instructions with scoring range set at 16 bit for optimized performance.

***GASAL2*** GASAL2 is a recently developed GPU implementation for short read DNA analysis that performs pairwise alignments. The authors of GASAL2 have comprehensively demonstrated that GASAL2 is the fastest GPU implementation for pairwise alignments [16]. Hence, to avoid redundancy, we only use GASAL2 among GPU libraries for performance comparison. GASAL2 has been optimized only for DNA sequences and does not support protein alignments.

***NVBIO*** NVBIO is a GPU based library developed by NVIDIA developers which provides multiple algorithms implemented for accelerating bioinformatics pipelines. For this paper we used the *proteinsw* program provided with the NVBIO library. Here, it needs to be considered that NVBIO has not been maintained for some time now and we found out that with the same scoring conditions, using the same dataset, NVBIO’s protein alignment output does not match with the well known Smith-Waterman implementations. An issue has been opened at their github page regarding this. The NVBIO-based experiments in this paper were performed assuming that the library still computes the complete scoring matrix, but because of a bug in the protein scoring function the results do not match with other libraries.

### Experimental data

To evaluate the performance of ADEPT against existing methods we identified use cases in genomics and proteomics that require performing large numbers of pairwise alignments. For each of these use-cases we obtained real world datasets, which were then processed to form three curated sets of Query and Reference sequences. Below we discuss in detail the data generation process for DNA and Protein evaluation datasets.

#### DNA Data

For the alignment of DNA sequences, we used a set of 29 million FASTQ reads, of lengths from 150 to 300, from the SYNTH64 dataset [36] as the query sequences. For the reference sequence set, we used a collection of 283842 contigs assembled from the reads, using the MetaHipMer [19] assembler. The sequences in the resulting query and reference sets were then binned based on the length of the query sequence to obtain three different datasets i.e. DNA-1, DNA-2 and DNA-3. Details of these datasets are available in Table 1.

**Table 1:** This table shows features of three datasets that were generated using a UPC++ implementation of MetaHipMer assembler for evaluating performance for DNA based applications

Dataset	Query Set		Reference Set		Total Alignments
	Min. Size	Max. Size	Min. Size	Max. Size	
DNA-1	150	200	99	779	31,071,476
DNA-2	201	250	99	979	8,892,748
DNA-3	251	300	99	1,131	16,308,186

#### Protein data

For the alignment of protein sequences, we use a curated (a combination of automatic and manual curation) dataset called SCOPe (Structural Classification of Proteins - extended) [37]. The current version of this dataset (2.07) contains around 244k proteins, of which we select the unique 77,040. The pairwise alignments are constructed within a protein family identification pipeline [38]. In this pipeline, a set of candidate pairs are filtered and passed to the aligner to obtain various alignment information. The number of pairs filtered by this pipeline is 54.5 million. The obtained alignment information is then used to construct the protein similarity network.

For all the pairwise alignments we assumed the longer sequence is the reference and the shorter sequence is the query. These sequences were then binned based on the length of the query sequences into three different datasets: Protein-1, Protein-2 and Protein-3. Details are provided in Table 2.

**Table 2:** This table shows the features of three datasets that were generated using most recent version of PASTIS for evaluating performance for protein based applications

Dataset	Query Set		Reference Set		Total Alignments
	Min. Size	Max. Size	Min. Size	Max. Size	
Protein-1	20	200	200	1,664	31,846,093
Protein-2	20	400	400	1,664	38,610,219
Protein-3	20	600	600	1,664	12,148,680

### Comparison with existing methods

For each of the above discussed datasets we performed three experiments to evaluate ADEPT’s performance against existing approaches. First we compare the Giga Cell Updates Per Seconds (GCUPS) for each approach, which was done by running all the methods in only forward scoring phase to obtain only the highest score. Then we compute the total cells (of the DP table) that were processed for that dataset and divide that by the total runtime, which is given by:

6 $$ \text{GCUPS} = \frac{\text{Total Cells}}{\text{Forward Scoring Time}}  $$

In the second experiment, we turn on the reverse scoring phase for all the algorithms (algorithms which do not support a reverse scoring phase were omitted from this experiment) and evaluate the total execution time for obtaining the score, start and end positions of the optimal alignment.

Finally, we repeat the above experiments by running all of the algorithms on complete CPU and GPU nodes of the Cori Supercomputer [39] to evaluate the ability of these implementations to support multiple numbers of GPUs, as is expected by high-performance bioinformatics pipelines.

### Experimental conditions

For all the CPU runs we made use of the Cori Supercomputer’s Haswell nodes [40], each of which consists of two sockets of Intel Xeon Processor E5-2698 v3, operating at 2.3 GHz with 16 CPU cores each, with a total of 32 cores per node. All the CPU libraries were built using GCC version 8.3.0 with optimizations turned on. For GPU runs we made use of the Cori Supercomputer’s GPU nodes [41], each of which consists of eight NVIDIA V100 GPUs. Each V100 GPU consists of 16 GB of Global Memory, 96KB of Shared Memory/L1 cache per SM and 6144KB of L2 cache in total. For computations, each V100 GPU contains a total of 80 Streaming Processors (SMs) and 64 FP32 units, giving a total of 5,376 computing cores. The GPU libraries were built using CUDA version 10.2.89.

For DNA alignments we used the same scores for all algorithms, i.e. match-score of 6, mismatch penalty of 4, gap open penalty of 4 and gap extension penalty of 1. For Protein alignments we used the Blosum 62 matrix [31], with a gap open penalty of 6 and gap extension penalty of 1. Typically, ADEPT’s driver determines the batch size at runtime but for these experiments we fixed the batch size to 20,000 since that provides optimal performance for the V100 GPUs. The batch size is a user configurable parameter.

### Performance on DNA alignments

**Fig. 8:**
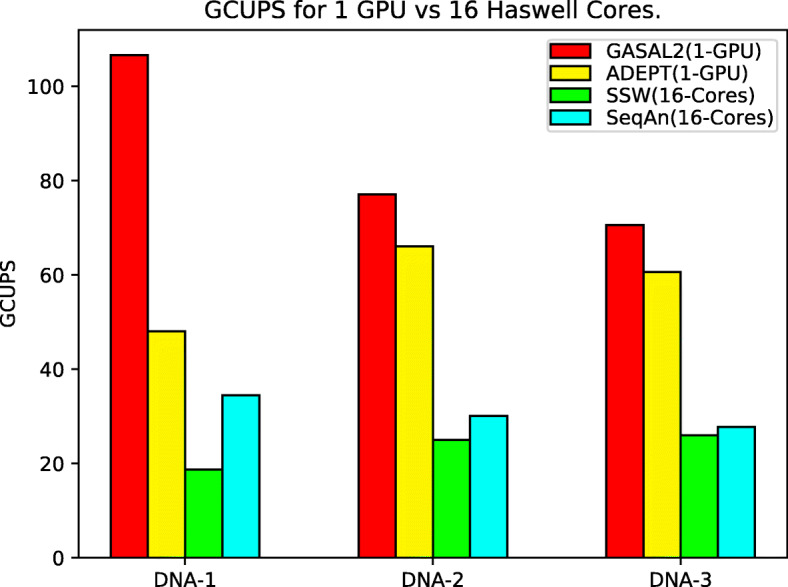
This figure shows total GCUPS (higher is better) for each algorithm when processing the DNA datasets in Table 1 using 1 CPU socket and 1 GPU

We first compared the total GCUPS for all algorithms for DNA alignment datasets. As discussed before, this was done by turning off the reverse scoring feature and only using one socket of a Haswell Node (16 CPU cores) for CPU libraries and one V100 GPU for GPU libraries. It can be observed in Fig. 8 that for shorter queries (DNA-1), the GASAL2 library out-performs all the algorithms, but for the remaining datasets where query lengths are longer, ADEPT starts performing better because of its intra-sequence parallelization strategy. This is because, as the size of the query increases, the number of elements that can be computed in parallel also increases, and this results in ADEPT closely matching GASAL2’s performance for the DNA-1 and DNA-2 datasets. For a single GPU, ADEPT gives a peak performance of about 66 GCUPS.

**Fig. 9:**
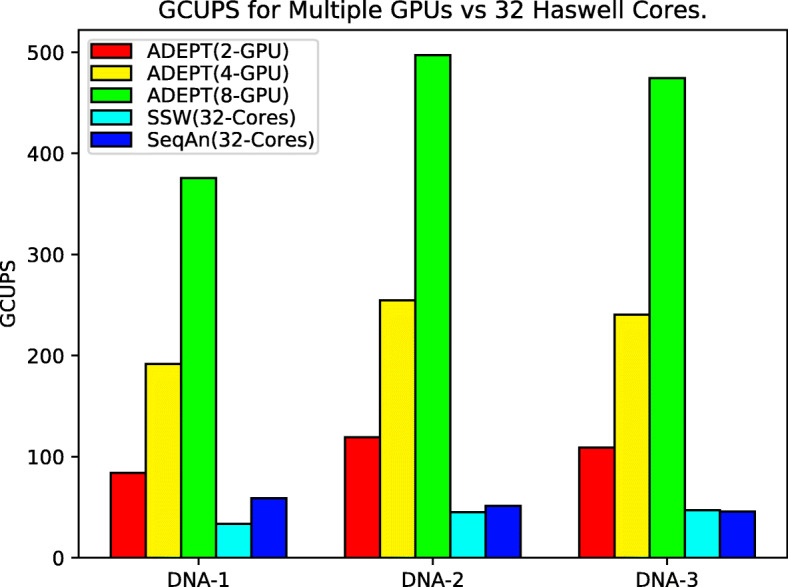
This figure shows total GCUPS (higher is better) for each algorithm when processing the DNA datasets in Table 1 while using complete nodes

To evalute the high-performance computational capability for large scale systems, we repeated the above runs, but this time for the CPU libraries we used all the available CPU cores on a Cori Haswell node (32 CPU cores). And for GPU codes, we performed analysis for 2 GPUs, 4 GPUs and full node runs with all 8 GPUs. GASAL2 does not have support for multiple GPUs, hence it was not included in these experiments. It can be observed in Fig. 9 that ADEPT scales quite well for an increasing number of GPUs and can provide a peak node performance of 497 GCUPS. An overall, node-to-node speedup of 11x and 10x was achieved over the SSW library and Seqan library respectively.

To compare overall performance, we turned on the reverse scoring feature for all algorithms so that each can compute the start position of the alignment as well. The Seqan benchmarking suite did not have an option of obtaining the start position using reverse scoring and instead performs a complete traceback which is a very slow, so in the interests of fairness, we did not include Seqan in these experiments. These experiments were again performed using a single socket (16 CPU cores) and a single GPU first and then repeated for full nodes to evaluate their ability for large scale bioinformatics tools.

**Fig. 10:**
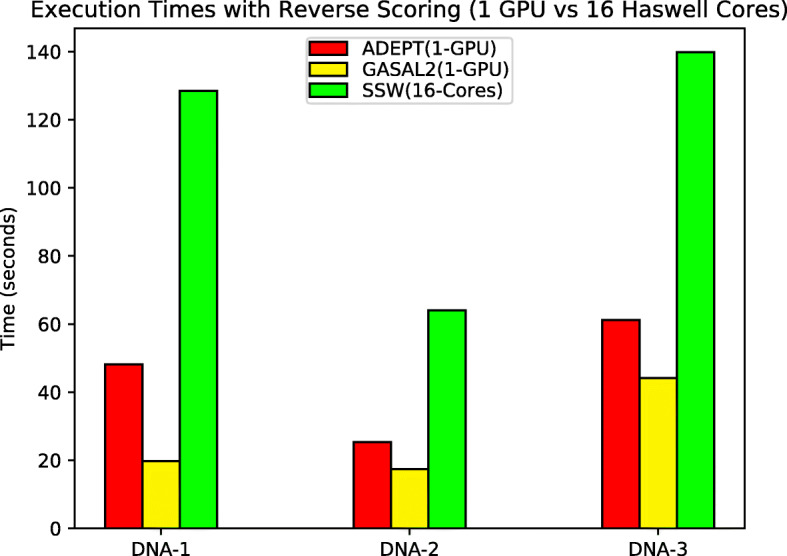
Total execution times (lower is better) for each algorithm when processing the DNA datasets in Table 1 with reverse scoring turned on, when using single CPU socket and single GPU

**Fig. 11:**
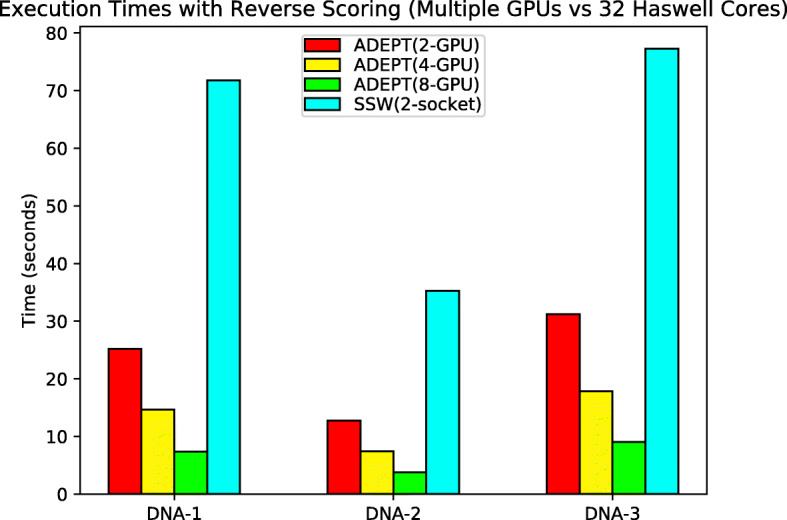
Total execution times (lower is better) for each algorithm when processing the DNA datasets in Table 1 with reverse scoring turned on, when using complete nodes

It can again be observed in Fig. 10, that GASAL2 performs better for the DNA-1 dataset where query lengths are limited to only 200 bases long. As we move onto the datasets with longer query sequences, ADEPT’s intra-sequence approach starts catching up owing to increased fine-grained parallelism. For full node runs we did not include GASAL2 because it does not provide multi-GPU support. Figure 11 shows that ADEPT out-performs the SSW-Library by about 10x in node-to-node comparisons and scales well for an increasing number of GPUs, even with the reverse scoring phase turned on.

### Performance on protein alignments

**Fig. 12:**
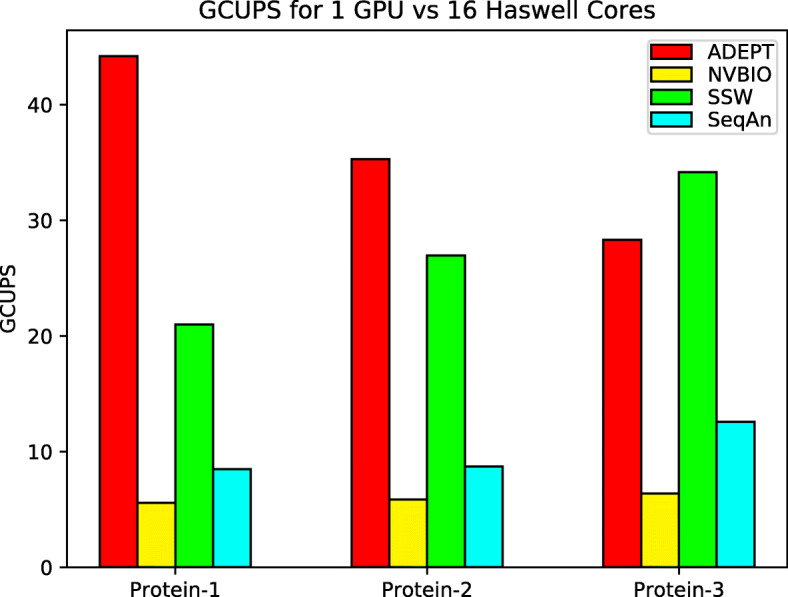
This figure shows total GCUPS (higher is better) for each algorithm when processing the protein datasets in Table 2 using 1 CPU socket and 1 GPU

**Fig. 13:**
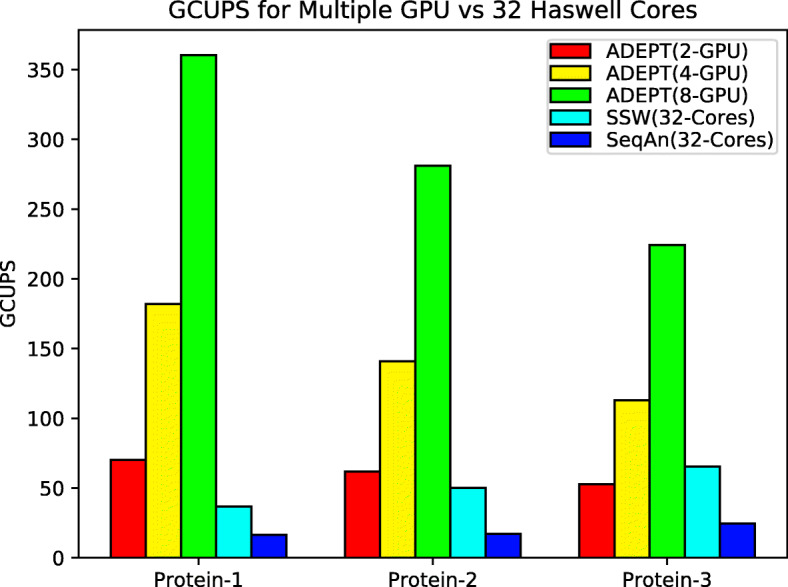
Total GCUPS (higher is better) for each algorithm when processing the protein datasets in Table 2 while using complete nodes

We repeated the same experiments for Protein datasets; for these experiments we do not include GASAL2 library because it does not support protein alignments. Figure 12 shows that for single GPU ADEPT out-performs NVBIO by about 8x for the Protein-1 dataset and consistently performs better than NVBIO for the remaining datasets. With CPU Libraries utilizing 16 CPU cores, ADEPT out performs them for the Protein-1 and Protein-2 dataset but for the Protein-3 dataset the SSW-Library catches up. This is because the SSW-Library uses a heuristic based approach and performs less work overall, so with increasing sequence lengths we see an improvement in the performance of the CPU Libraries. However, the true potential of the GPU accelerated approach is observed when performing node-to-node analysis, as typically an HPC cluster has more GPUs per node than CPU sockets. In Fig. 13, we perform a node-to-node analysis with the CPU libraries making use of all the 32 CPU cores on each Haswell node of the Cori Supercomputer, while ADEPT utilizes all 8 GPUs available on a Cori GPU node. It can be observed that for complete node-to-node analysis, ADEPT dominates and gives a peak performance of 360 GCUPS when using all eight GPUs. Since NVBIO does not provide a driver program like ADEPT to support multiple GPUs, we did not include it in these experiments.

**Fig. 14:**
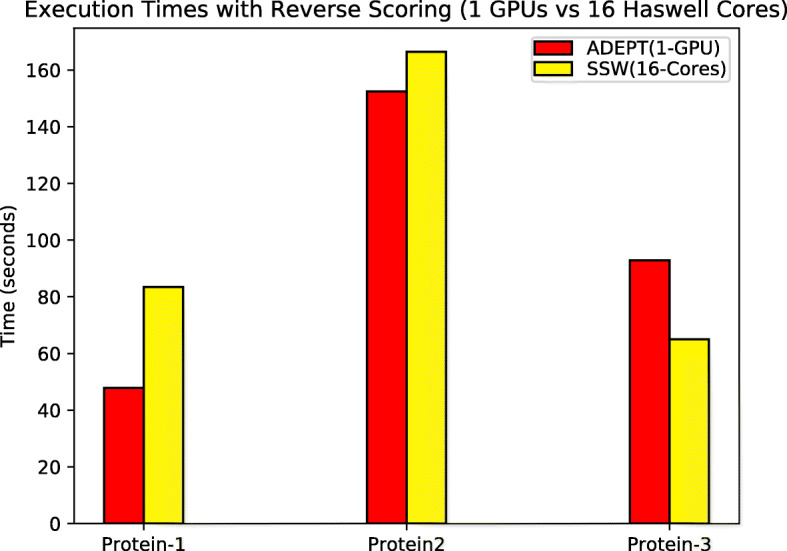
Total execution times (lower is better) for each algorithm when processing the protein datasets in table 2 with reverse scoring turned on, using single CPU socket and single GPU

To evaluate the performance for protein alignments with reverse scoring phase turned on, we compared only the SSW-Library and ADEPT because the Seqan test suite and NVBIO do not have the reverse scoring feature. For single socket (16 CPU core) vs single GPU comparison, ADEPT out-performs SSW for the Protein-1 and Protein-2 datasets (Fig. 14). But for the larger sequence dataset (Protein-3), the SSW Library catches up owing to its heuristic based scoring algorithm.

**Fig. 15:**
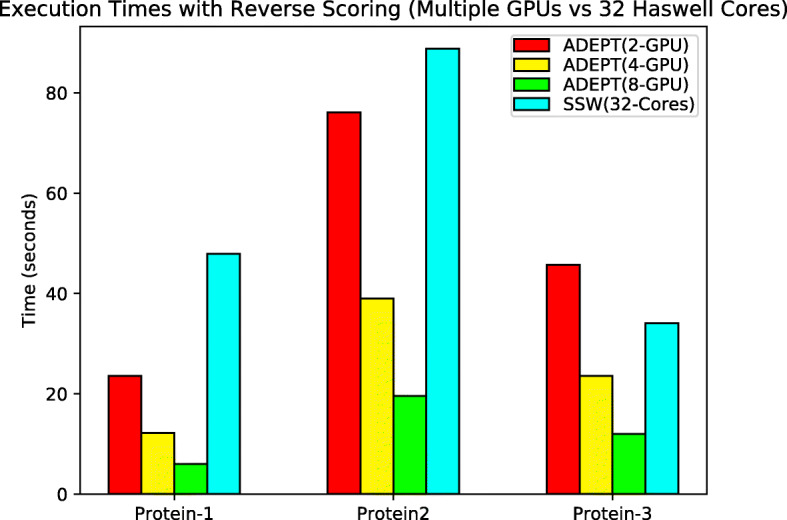
Total execution times (lower is better) for each algorithm when processing the protein datasets in Table 2 with reverse scoring turned on, using complete nodes

But for node level analysis, ADEPT (Fig. 15) out performs SSW-Library by almost 8x.

## Application use cases

To demonstrate ADEPT-SW’s effectiveness in preparing large-scale bioinformatics software pipelines for GPU-heavy systems, we chose two applications from different domains. The first is MetaHipMer [19, 42], which is a large-scale metagenome assembly pipeline for performing denovo assembly of metagenomic data sets, and the second is PASTIS [38], a distributed protein network construction pipeline. ADEPT-SW was integrated into both these pipelines to accelerate the portion which makes use of the Smith-Waterman local alignment algorithm. Based on the design of these pipelines, different approaches were adopted to take advantage of the GPU-accelerated ADEPT-SW library.

### Use-case: metagenome assembly

**Fig. 16:**
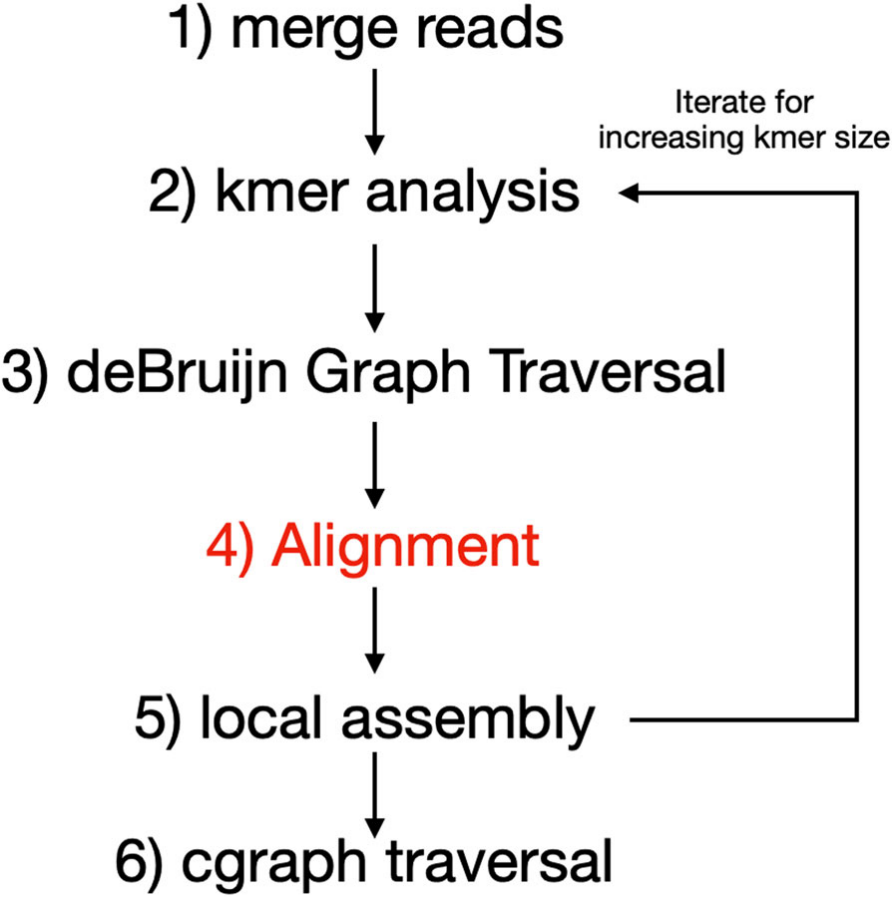
An overview of MetaHipMer pipeline. The alignment step (highlighted in red) makes use of Smith-Waterman alignments to map short reads to longer target sequences

MetaHipMer is a specialized version of a large scale denovo genome assembler HipMer [2]. Metagenome assembly involves processing a DNA dataset obtained from a microbial colony into a complete representation of the underlying genome. MetaHipMer has been designed for large scale supercomputers using a partitioned global address space (PGAS) programming model which enables it to run on a shared memory computer as well as a large-scale distributed machine. An overview of the MetaHipMer pipeline can be seen in Fig. 16. The fourth step highlighted in red is the alignment phase of MetaHipMer where the input reads are aligned against target sequences that have been built in the stages before. At the core of alignment step, MetaHipMer uses a CPU-based kernel of a Smith-Waterman Library called SSW [27], which has also been discussed before in section Results.

**Fig. 17:**
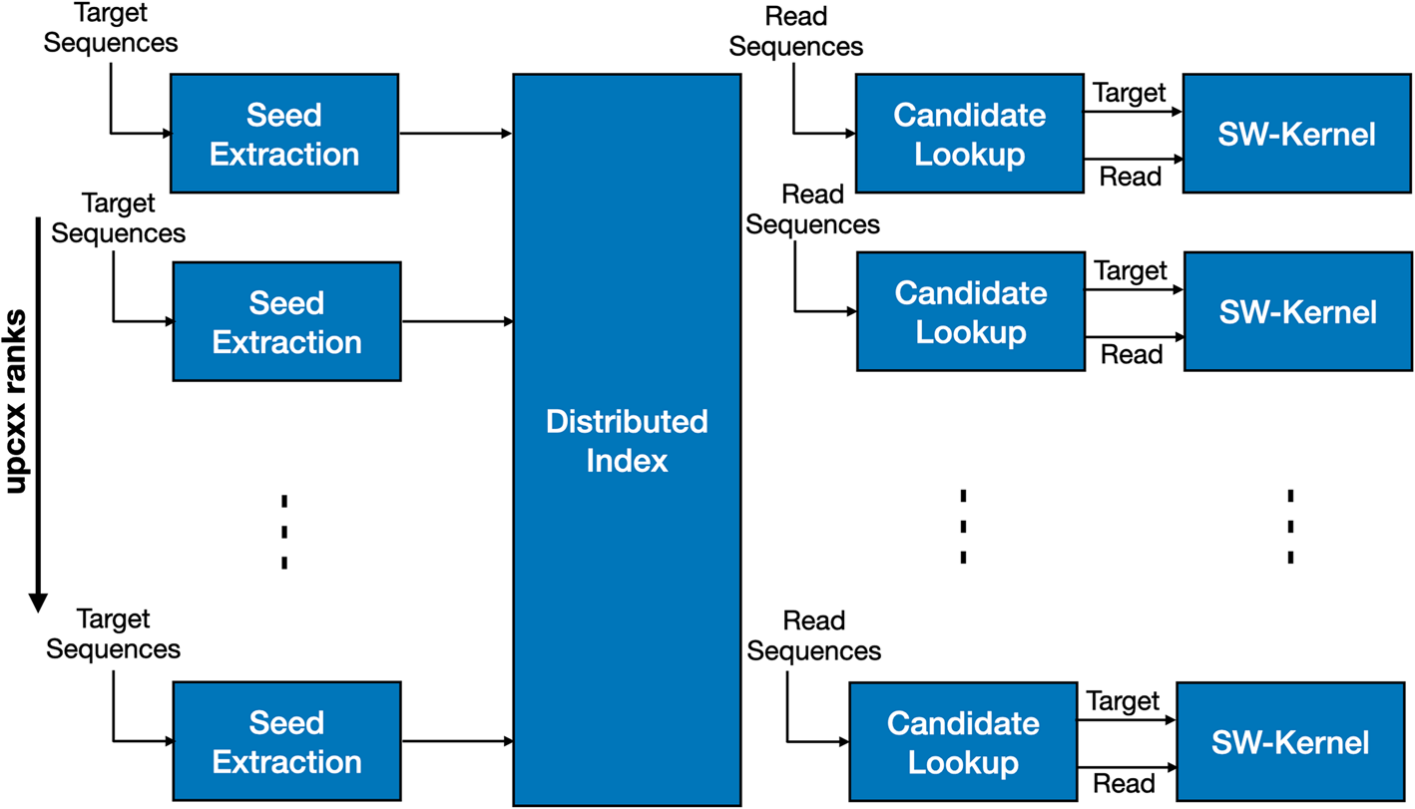
MetaHipMer’s alignment phase using the CPU Smith-Waterman Kernel. Each rank gets a set of target sequences which are used to construct a global seed index stored in shared memory. Each process then performs a lookup in the distributed seed index and obtains a set of possible target candidates. A pair of target and read candidates are then passed to the SSW Kernel

A simplified overview of the alignment phase of MetaHipMer can be seen in Fig. 17. MetaHipMer uses multiple processes on each node to take advantage of the underlying parallel hardware. Each process parses a set of reads independently, performs a candidate lookup in the distributed index and obtains a set of possible candidates to which that read might align. The target and read pair is then passed to SW-Kernel on the CPU to process the alignment.

#### ADEPT-SW integration

**Fig. 18:**
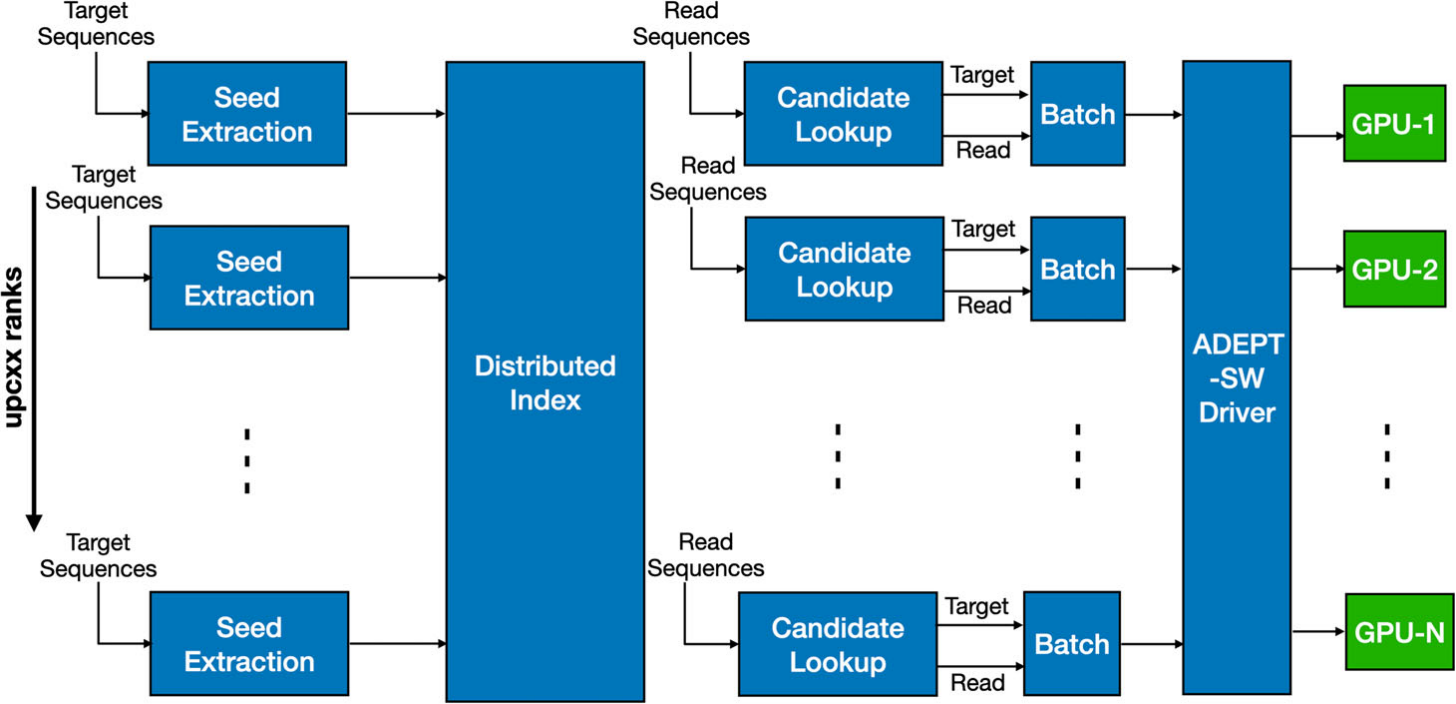
MetaHipMer’s alignment phase is modified so that instead of calling the CPU SSW kernel, the read and target pair is added to a batch. This batch is then passed to the ADEPT-SW driver, which then takes over and performs a CPU-process to GPU mapping and balances the load so that each GPU is performing an equal amount of work

We modified the MetaHipMer pipeline so that instead of performing a SW alignment immediately after the lookup, it adds it to a batch of alignments. Once the batch size is large enough, a call is made to the ADEPT-SW kernel which then takes control. The ADEPT-SW kernel detects the number of available GPUs and the number of processes running on the CPU and then performs a mapping from CPU to GPU in a round-robin fashion. If the number of GPUs is smaller than the number of processes on the node, multiple processes may be mapped to a same GPU. During the mapping the ADEPT-SW kernel ensures that if a GPU is shared by more than one process, the global memory of the GPU is divided among the processes such that there is no overlap. The ADEPT-SW driver then launches GPU kernels as shown in Fig. 3. The modified version of the mteaHipMer alignment phase can be seen in Fig. 18.

#### Results

For this study a UPC++ based version of MetaHipMer was used. All the experiments were performed on Cori GPU Nodes [41]; each GPU node consists of eight NVIDIA V100 GPUs and two sockets of Intel Xeon Gold 6148 (Skylake) processors, where each socket consists of 20 CPU cores, making a node total of 40 CPU cores. We used ArcticSynth dataset for these experiments [42].

**Fig. 19:**
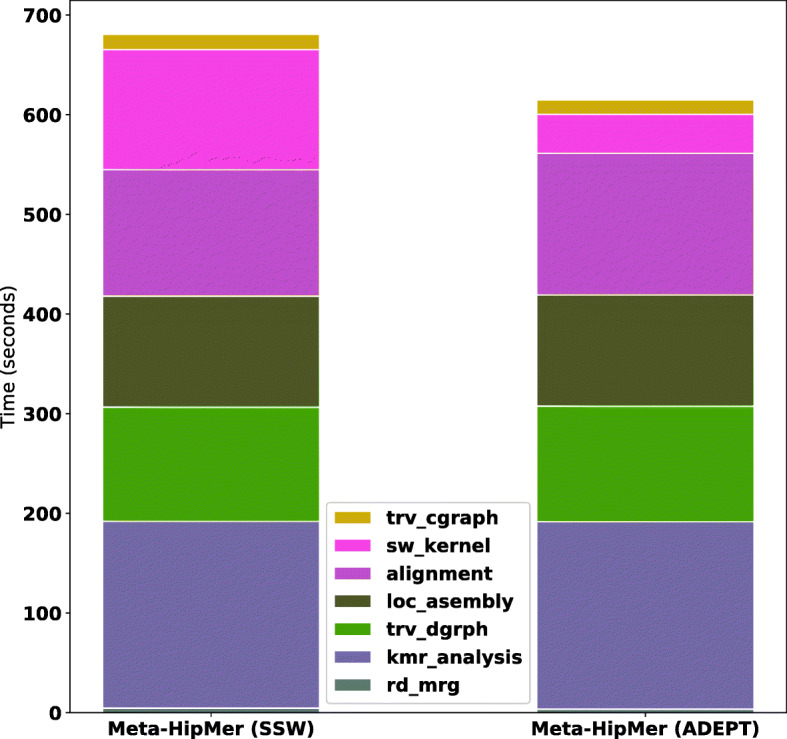
This figure shows the runtime breakdown of MetaHipMer pipeline with and without ADEPT-SW integration while processing the ArcticSynth dataset [42]. It can be observed that Smith Waterman (sw_kernel in legend) portion of pipeline speeds up by 2.9x giving alignment portion a speedup of 36%. Overall, MetaHipMer pipeline shows 10% performance improvement with ADEPT-SW integration

Figure 19 shows the performance comparison between MetaHipMer and ADEPT integrated MetaHipMer. The Smith-Waterman portion improves by 2.9x which gives the alignment phase a performance boost of 36% and an overall pipeline performance improvement of 10% was observed for a single node run.

### Use-case: protein similarity graph construction

**Fig. 20:**
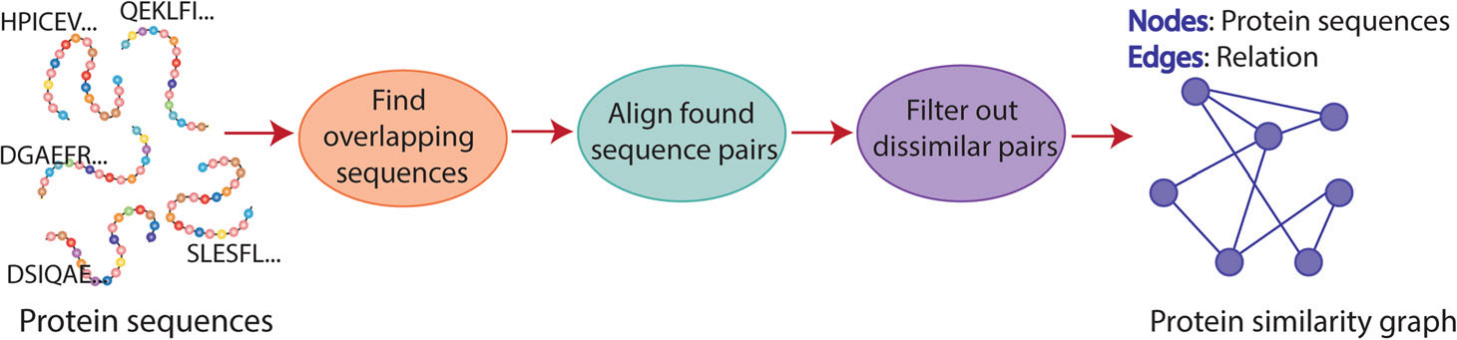
Components of PASTIS

PASTIS [38] is a distributed-memory software for performing large-scale protein similarity search. Its goal is to facilitate the fast construction of huge protein similarity graphs, which are then usually utilized in a clustering phase to discover similar proteins. It encodes protein sequence information within distributed sparse matrices and relies on sparse matrix operations to discover candidate protein sequence pairs for further alignment. The main components of PASTIS are illustrated in Fig. 20. The alignment of candidate protein sequence pairs is performed after discovering overlapping protein sequence pairs and it constitutes one of the most computationally expensive components. In PASTIS, the alignments performed by a process are independent from the alignments performed by other processes, i.e., they are performed locally by each individual process. Hence, although ADEPT-SW does not support distributed memory parallelism, PASTIS can still make use of it as the alignment component is local to each process.

PASTIS needs certain information regarding the aligned sequence pairs in the construction of the protein similarity graph. These information include average nucleotide identity, raw alignment score, coverage, etc. The alignments that do not meet certain criteria are eliminated and they are not included in the protein similarity graph. Note that each aligned sequence pair corresponds to a possible edge in the protein similarity graph, where the weight of the edge signifies the strength of similarity between the aligned sequence pair. For that purpose, PASTIS can make use of either average nucleotide identity or normalized raw alignment score.

#### Adept-sw integration

PASTIS originally relies on Seqan C++ library (The Library for Sequence Analysis) [26] for alignment. Its representation of protein sequences via matrices allows easy integration. In the matrix produced by PASTIS at the end of the overlap detection component in Fig. 20, each process stores a distinct rectangular block of this matrix. Each non-zero element in this block signifies an alignment to be executed by the aligner. Hence, at each process, we traverse these elements in batch by storing them in necessary structures required by ADEPT. Then, the control passes to ADEPT driver component, which performs alignments across all the GPUs available on the node and produces output information required by PASTIS. Specifically, PASTIS uses the raw score and coverage of each alignment computed by ADEPT in the formation of the protein similarity graph.

#### Results

In the evaluation of PASTIS, we follow a similar setting to that of MetaHipMer. The experiments are performed on a single node of Cori GPU Nodes [41]. We used SCOPe (Structural Classification of Proteins - extended) [37] dataset for PASTIS. For the alignment component, we tested out Seqan’s Smith-Waterman alignment algorithm and ADEPT-SW. We turned off trace-back component of Seqan, as is the case for ADEPT-SW. For PASTIS, we used a k-mer size of 6 and 10 substitute k-mers. This results in around 31.2 million sequence pairs that need to be aligned by Seqan or ADEPT-SW.

**Fig. 21:**
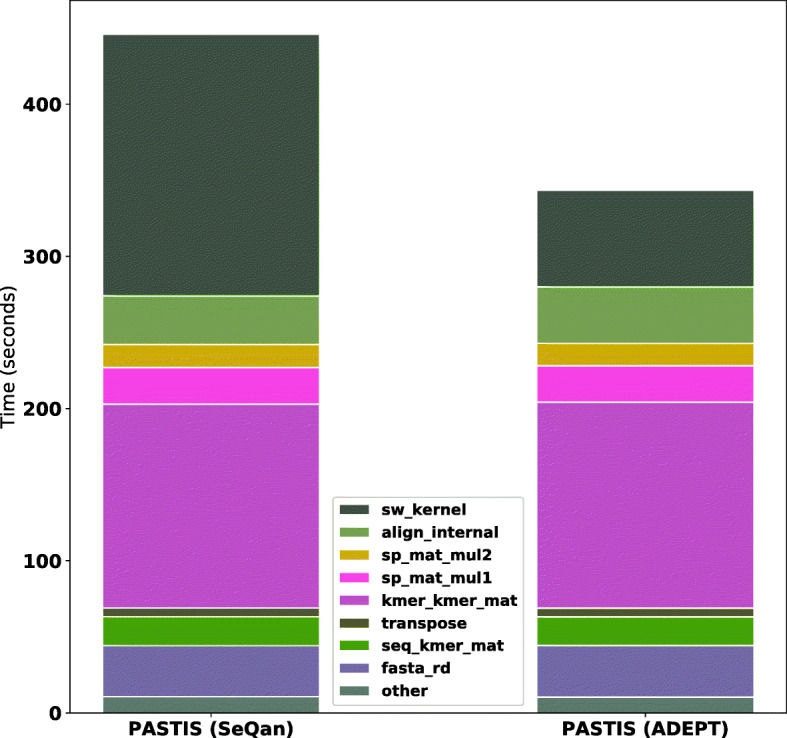
This figure shows the runtime breakdown for PASTIS pipeline with and without ADEPT-SW integration while processing the SCOPe dataset [37]. PASTIS with ADEPT integeration sees about 2.7x reduction in Smith Waterman alignments duration (sw_kernel in legend). Overall 30% improvement in the performance of PASTIS is observed

Figure 21 shows the runtime dissection of PASTIS when run with Seqan and ADEPT-SW. The alignment constitutes almost half of the execution time of PASTIS when it is run with Seqan (indicated with the legends sw_kernel and align_internal). A comparison of performance in Fig. 21 shows that PASTIS with ADEPT integration performs Smith Waterman alignments about 2.7x faster than Seqan (compare the values for legend sw_kernel) and improves the performance of overall pipeline by 30%.

## Conclusions

Aligning two biological sequences is fundamental to a majority of computational pipelines in modern bioinformatics. Applications such as homology, the study of proteins and genes, protein database search, protein clustering, de novo genome sequencing, metagenome analysis and functional genomics and proteomics, frequently make use of sequence alignment algorithms at their core. Optimal sequence alignment algorithms like Smith-Waterman are based on dynamic programming approaches which makes them computationally intensive and difficult to parallelize owing to their convoluted dependencies. With the move of supercomputing facilities towards heterogeneous architectures, a lot of effort is being made to port existing bioinformatics workflows to the new architectures. In this process, sequence alignment algorithms have emerged as a computational as well as an implementation challenge. Existing GPU libraries employ domain and application specific strategies for optimizing an algorithm’s performance on GPUs, but such strategies are not generic and have restricted use-cases. This called for a domain independent strategy that targeted the hardware for speed and not the data, similar to the methods widely available for CPUs.

As a way forward through this problem, we have introduced ADEPT in this paper, which is a domain independent strategy for sequence alignment that leverages the GPU’s architecture for accelerating dynamic programming based sequence alignment algorithms. We introduced a novel data structure to tackle the inter-thread dependencies and utilized register-to-register data transfers for efficient communication between CUDA threads. We demonstrated the performance of this strategy by implementing the Smith-Waterman algorithm, an optimal local alignment algorithm, and comparing its performance with similar approaches for CPU SIMD units and the existing fastest GPU implementations available for DNA and Protein alignment. ADEPT has shown performance that either closely matches or is better than existing CPU and GPU methods. We used a variety of real world datasets, ranging from proteins and DNA sequences with varying reference and query lengths, to rigorously evaluate the performance of ADEPT. For DNA and protein use-cases, ADEPT demonstrated a peak speed up of 10x and 7x respectively in node-to-node analysis against CPU libraries, while out-performing or closely matching the performance of existing GPU libraries. We also demonstrated the usability of ADEPT by integrating it into existing bioinformatics software pipelines and demonstrate a 10% and 30% boost in performance of MetaHipmer and PASTIS softwares.

ADEPT is a generic strategy and can be easily extended for other dynamic programming-based sequence alignment algorithms such as those used for global and semi-global alignments. We hope ADEPT’s capability of exploiting a GPU’s architecture to provide a unified solution to GPU sequence alignment, its ability to scale across multiple GPUs and ease of use will enable it to take a central role in future high-performance bioinformatics application development and porting.

## Availability and requirements

**Project Name:** ADEPT based implementation of Smith-Waterman**Project home page:** https://github.com/mgawan/GPU-BSW**Operating System:** Linux**Programming Language:** C++, CUDA**Other requirements:** CUDA 10 or later**License:** OSS**Any restrictions to use by non-academics:** use the included license

## Data Availability

Software developed as part of this manuscript has been made available at the repository above with an open source license. Openly available datasets were used for evaluation of this software, original sources of datasets have been referenced in the section Experimental data.

## Abbreviations

CUDA: Compute Unified Device Architecture
GPU: Graphics Processing Unit
CPU: Central Processing Unit
GCUPS: Giga Cell Updates Per Seconds
DNA: Deoxyribonucleic acid
SIMD: Single Instruction Multiple Data
ASCII: American Standard Code for Information Interchange
